# Appropriateness of aspirin prescribing for primary and secondary prevention of cardiovascular disease in type 2 diabetes in different care settings

**DOI:** 10.1007/s11845-021-02649-5

**Published:** 2021-06-22

**Authors:** Shi Ying Tan, Heather Cronin, Stephen Byrne, Adrian O’Donovan, Antoinette Tuthill

**Affiliations:** 1 School of Medicine, University College Cork, College Road, Cork, Ireland; 2 Department of Endocrinology and Diabetes, Cork University Hospital, Wilton, Cork, Ireland; 3 School of Pharmacy, University College Cork, College Road, Cork, Ireland; 4 Elmwood Primary Care Centre, Frankfield, Douglas, Cork Ireland

**Keywords:** Cardiovascular disease, Diabetes mellitus, Primary prevention

## Abstract

**Background:**

Type 2 diabetes is associated with an increased cardiovascular risk. Use of aspirin has been shown to be of benefit for secondary prevention of cardiovascular disease in patients with type 2 diabetes; benefits in primary prevention have not been clearly proven.

**Aims:**

This study aims to (a) determine if aspirin is prescribed appropriately in type 2 diabetes for primary or secondary prevention of cardiovascular disease (CVD) and (b) evaluate whether there are differences in aspirin prescribing according to where people receive their care.

**Design:**

Cross-sectional study

**Methods:**

The medical records of individuals with type 2 diabetes aged over 18 years and attending Elmwood Primary Care Centre and Cork University Hospital Diabetes outpatient clinics (*n* = 400) between February and August 2017 were reviewed.

**Results:**

There were 90 individuals exclusively attending primary care and 310 persons attending shared care. Overall, 49.0% (*n* = 196) of those were prescribed aspirin, of whom 42.3% were using it for secondary prevention. Aspirin was used significantly more in people attending shared care (*p* < 0.001). About 10.8% of individuals with diabetes and CVD attending shared care met guidelines for, but were not prescribed aspirin.

**Conclusion:**

A significant number of people with type 2 diabetes who should have been prescribed aspirin for secondary prevention were not receiving it at the time of study assessment. In contrast, a substantial proportion who did not meet criteria for aspirin use was prescribed it for primary prevention.

## Introduction

Type 2 diabetes mellitus is a chronic condition associated with multiple complications and comorbidities. If poorly managed, diabetes can lead to functional limitations, disability, reduced quality of life, and life expectancy. According to the Irish CODEIRE study (2006), approximately 10.0% of the Ireland’s national healthcare budget was spent on diabetes with nearly half that amount due to hospitalizations attributable to the microvascular and macrovascular complications associated with the disease. A large proportion of these admissions could be prevented with adequate blood glucose control and mitigating overall vascular risk. [1].

In 2012, diabetes alone claimed 1.5 million lives worldwide [2] with the greatest mortality due to cardiovascular disease (CVD). [3] Persons with type 2 diabetes have a two to fourfold increased risk of developing CVD, [4] mainly due to increased arachidonic acid metabolism and thromboxane A_2_ synthesis, resulting in augmented platelet aggregation and a pro-inflammatory substrate. Low-dose aspirin inhibits platelet cyclooxygenase 1, the key enzyme responsible in thromboxane A_2_ synthesis, subsequently preventing thrombus formation. [5] .

Multiple studies support aspirin prescription for secondary prevention of cardiovascular events as it reduces mortality, and morbidity from non-fatal vascular events (i.e., stroke or myocardial infarction), and total vascular events, respectively. [6, 7] With regard to primary prevention, historically persons with type 2 diabetes without a prior history of CVD were recommended to commence on aspirin, as diabetes is considered as a CVD risk equivalent. [8] However, the benefits of aspirin are ambiguous and recent studies including the ARRIVE, ASPREE, and in particular ASCEND trials have resulted in changes to guidelines. [9–11].

Generally, the guidelines for aspirin use in primary prevention are contradictory. The American Diabetes Association (ADA) has recommended aspirin use as primary and secondary prevention of CVD in diabetes since 1997 [12] and has updated their guidelines several times with minor modifications. In the recent 2020 guidelines, the advice given is that low-dose aspirin (75–162 mg/day) should:

1. Be commenced for secondary prevention in persons with previous angina, MI, vascular bypass procedure, stroke or transient ischaemic attack (TIA), claudication, and/or peripheral vascular disease (PVD);
2. Be recommended for primary prevention in persons who have more than 10% increased 10-year cardiovascular risk, including age ≥ 50 years old who have one or more major risk factors, such as family history of CVD, hypertension, smoking, dyslipidaemia, or albuminuria.

The American Diabetes Association stated that individuals should not be on aspirin therapy if they are younger than 21 years old, or have any specific contraindications, which include aspirin allergy, bleeding tendency, recent gastrointestinal bleeding, and clinically active hepatic disease. Clinical judgment on a case-to-case basis is required for low-risk patients, such as those aged < 50 years with one or more risk factors, older patients with no risk factors, or those with 5–10% 10-year cardiovascular risk. [13].

As recent evidence demonstrates that there are limited benefits to aspirin use in the primary prevention setting, both the 2016 European Guidelines on Cardiovascular disease prevention in clinical practice and the latest practical guide to integrated type 2 diabetes mellitus by Irish College of General Practitioners (ICGP) in 2016 [14] have altered their guidelines and do not support routine aspirin use in type 2 diabetes without CVD.

This study aims to investigate the appropriateness of aspirin prescribing in type 2 diabetes by (a) comparing aspirin prescribing in a primary and secondary care setting and (b) determining whether aspirin is being used for primary or secondary prevention of CVD.

## Methods

### Study design, population, and sample

This was a cross-sectional study based on medical records of persons monitored on an outpatient basis. All individuals with type 2 diabetes who attended the Elmwood Primary Care Centre and those attending the diabetes outpatient clinics in Cork University Hospital, a large tertiary referral hospital, from February 2017 to August 2017 were eligible for recruitment. Adults aged over 18 years diagnosed with type 2 diabetes before January 2016 and had at least a period of 3-month follow-up were included. Individuals who failed to attend care for more than a year, those diagnosed with pancreatic insufficiency, cystic fibrosis-related diabetes, and those allergic to aspirin were excluded from the study.

A data collection form was used to record individuals' demographic data (date of birth, current and age of diagnosis, gender, ethnicity), anthropometric data (weight and height), smoking status, and care status (care by GP only or shared care). Blood pressure and the most recent laboratory data, such as HbA_1_c, total cholesterol, high-density lipoprotein (HDL) cholesterol, low-density lipoprotein (LDL) cholesterol, and urine albumin:creatinine ratio were documented. In addition, diabetes associated comorbidities (e.g., hypertension, hypercholesterolaemia, ischaemic heart disease, MI, cerebrovascular accident, peripheral artery disease, presence of retinopathy or neuropathy, history of peptic ulcer disease and gastritis), medications prescribed, and use of aspirin or other oral anticoagulation drugs were included in the data collection.

The ICGP’s guidelines (adapted from both ADA's and NICE’s guidelines) were used as the standard. Target levels for HbA1c were ≤ 53 mmol/mol (≤ 7.0%) for majority of individuals, and < 48 mmol/mol (< 6.5%) for those without CVD, and for those on lifestyle modification or metformin use only. Hypertension was defined as either a systolic blood pressure of > 140 mmHg or diastolic pressure of > 80 mmHg, or if the patient was taking antihypertensive medications. [14]^.^

Individuals were recorded as having CVD if they had a history of either ischaemic heart disease, MI, percutaneous coronary intervention, coronary artery bypass grafting, stroke and/or TIA, and PVD. The 10-year cardiovascular risk for persons with type 2 diabetes without CVD was estimated using the UK Prospective Diabetes Study (UKPDS) risk stratification tool. [15] Individuals who were on aspirin for CAD, stroke/TIA, and PVD were classified under secondary prevention. All others were considered as primary prevention.

### Statistical analysis

Statistical analyses were done using IBM SPSS Statistics version 21.0. Descriptive statistics were used to describe the characteristics of aspirin use among individuals with type 2 diabetes in Cork. Independent *t*-test or Mann–Whitney test was used to compare means between individual groups (primary care and shared care, aspirin and non-aspirin users, primary prevention, and secondary prevention). The chi-square test or Fisher’s exact test was used to determine the association between characteristics and these groups. In all hypothesis tests, two-sided tests were used and *p* < 0.050 was considered statistically significant. Individual patient data was not identifiable from the final analysis. The Clinical Research Ethics Committee (CREC) of the Cork Teaching Hospitals approved this study.

## Results

### Characteristics of the study population

A total of 400 individuals with type 2 diabetes were recruited into the study, with 90 of those exclusively attending primary care and 310 persons under shared care. There was a male predominance in both groups (61.1% in primary care and 57.7% in shared care). Individuals attending shared care had poorer control of their diabetes, more co-morbidities, and complications. Aspirin was used significantly more in those under shared care (*p* < 0.001). Characteristics of both groups are shown in Table 1.

**Table 1:** Clinical characteristics of participants recruited from primary and secondary care settings

Variable	*n*	Total	GP care only (*n* = 90)	Shared care (*n* = 310)	*p* value
Mean age (SD), years	400	64.3 (13.21)	62.3 (14.62)	64.8 (12.74)	0.113
Males, *n* (%)	400	234 (58.5)	55 (61.1)	179 (57.7)	0.568
Mean weight (SD), kg	394	88.86 (20.24)	89.19 (18.96)	88.78 (20.60)	0.868
Mean body mass index (SD), kg/m^2^	346	31.59 (6.60)	31.35 (6.09)	31.66 (6.76)	0.705
Median duration of diabetes (IQR), years	400	9.0 (6.0–15.0)^†^	5.0 (4.0–8.0)^†^	11.0 (7.0–17.0)^†^	< 0.001‡
Smoking status, *n* (%)	400				0.001
Current		51 (12.8)	12 (13.3)	39 (12.6)	
Never		226 (56.5)	65 (72.2)	161 (51.9)	
Former		123 (30.8)	13 (14.4)	110 (35.5)	
Mean Clinical laboratory measurements (± SD)					
HbA1c (mmol/mol)	400	57.8 (16.03)	53.3 (16.85)	59.1 (15.57)	0.002
Patients without CVD or on diet/metformin use only	241	54.1 (15.40)	52.9 (17.59)	54.7 (15.23)	0.385
Remaining patients	159	63.3 (15.44)	55.9 (10.23)	53.8 (15.654)	0.102
Total cholesterol (mmol/L)	395	4.36 (2.00)	4.81 (1.30)	4.23 (2.15)	0.015
HDL cholesterol (mmol/L) Male Female	381 224 158	1.18 (0.32) 1.09 (0.28) 1.30 (0.33)	1.21(0.28) 1.14 (0.24) 1.33 (0.30)	1.17 (0.33) 1.07 (0.29) 1.29 (0.34)	0.238 0.132 0.532
LDL cholesterol (mmol/L) In CVD patients In non-CVD patients	368 109 259	2.33 (0.88) 2.04 (0.76) 2.45 (0.90)	2.71 (1.02) 2.18 (0.73) 2.90 (1.04)	2.22 (0.80) 2.00 (0.76) 2.31 (0.80)	< 0.001 0.338 < 0.001
Systolic blood pressure (mmHg)	398	134.3 (15.58)	132.2 (11.68)	134.9 (16.50)	0.084
Diastolic blood pressure (mmHg)	398	76.85 (10.09)	79.3 (9.17)	76.2 (10.24)	0.010
Therapeutic targets not achieved, *n* (%)					
Blood pressure	398	168 (42.2)	36 (40.4)	132 (42.7)	0.703
HbA_1c_ Patients without CVD or on diet/metformin use only (≥ 48 mmol/L or ≥ 6.5%) Remaining patients (> 53 mmol/L or > 7.0%)	400	271 (67.8) 153 (56.5) 118 (43.5)	49 (54.4) 41 (95.9) 8 (16.3)	222 (71.6) 112 (50.5) 110 (49.5)	0.002 0.009 1.000§
HDL Male (< 1.0 mmol/L) Female (< 1.3 mmol/L)	382	179 (46.9) 93 (52.0) 86 (48.0)	33 (37.9) 17 (51.5) 16 (48.5)	146 (49.5) 76 (52.1) 70 (47.9)	0.058 0.086 0.441
LDL In CVD patients (> 1.8 mmol/L) In non-CVD patients (> 2.5 mmol/L)	368	161 (43.8) 63 (39.1) 98 (60.9)	52 (60.5) 15 (28.8) 37 (71.2)	109 (38.7) 48 (44.0) 61 (56.0)	< 0.001 0.270 < 0.001
Past medical history *n* (%)					
Diagnosed hypertension	400	221 (55.3)	36 (40.0)	185 (59.7)	0.001
Dyslipidaemia	400	177 (44.3)	27 (30.0)	150 (48.4)	0.002
Coronary heart disease	400	86 (21.5)	20 (22.2)	66 (21.3)	0.850
Cerebrovascular accident	400	40 (10.0)	8 (8.9)	32 (10.3)	0.690
Peripheral vascular disease	400	16 (4.0)	0 (0.0)	16 (5.2)	0.028^§^
Peptic ulcer disease	400	11 (2.8)	3 (3.3)	8 (2.6)	0.716^§^
Gastritis	400	17 (4.3)	7 (7.8)	10 (3.2)	0.074^§^
Microvascular complications, *n* (%)					
Diabetic retinopathy	363	82 (22.6)	9 (10.6)	73 (26.3)	0.002
Diabetic neuropathy	400	82 (20.5)	10 (11.1)	72 (23.2)	0.012
Albuminuria	350				
Microalbuminuria Macroalbuminuria		75 (21.4) 21 (6.0)	9 (15.8) 1 (1.8)	66 (22.5) 20 (6.8)	0.137

^†^The distribution is skewed to the right

^‡^Mann–Whitney test

^§^Fisher’s exact test

### Aspirin use in a primary and secondary setting

Overall, nearly half (49.0%) of the participants in this study were prescribed aspirin. Out of the 204 non-aspirin users, 22 (10.8%) were previously on aspirin and 47 (23.0%) were on other antiplatelet/anticoagulant agents. Aspirin users were older (*p* < 0.001), had a diagnosis of diabetes for a longer period (*p* < 0.001), higher HbA_1c_ (*p* = 0.059), and higher systolic BP at their last clinic visit (*p* = 0.018). Aspirin users were also more likely to have diagnosed hypertension (*p* = 0.001) and dyslipidaemia (*p* < 0.001).

### Appropriate use of aspirin for primary and secondary prevention of CVD

**Fig. 1:**
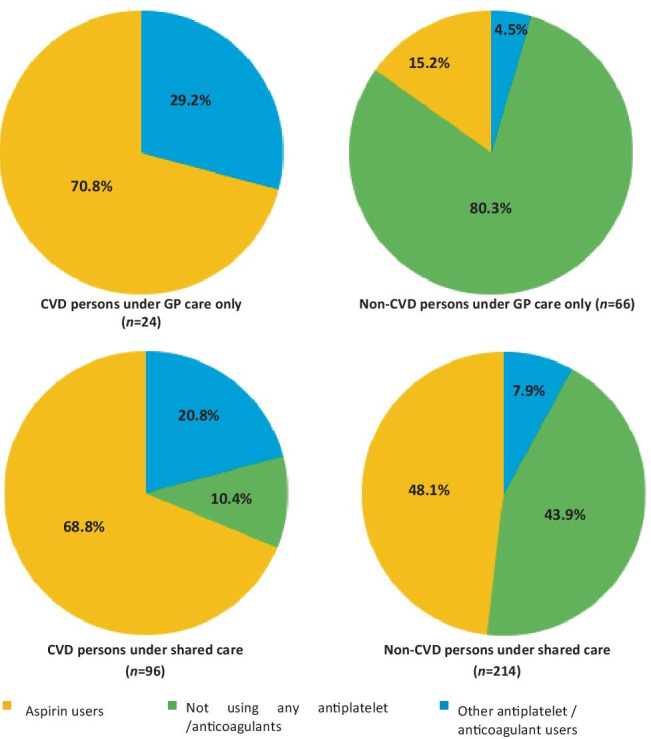
Appropriateness of use of aspirin in participants with type 2 diabetes attending primary or secondary care

All CVD patients under GP care were on aspirin or other antiplatelet/anticoagulant, while 10.4% of participants under shared care had CVD but were not on any antiplatelet or anticoagulant. On the contrary, 15.2% and 48.1% of persons under GP care and shared care respectively were on aspirin despite never having a diagnosis of CVD (Fig. 1).

**Fig. 2:**
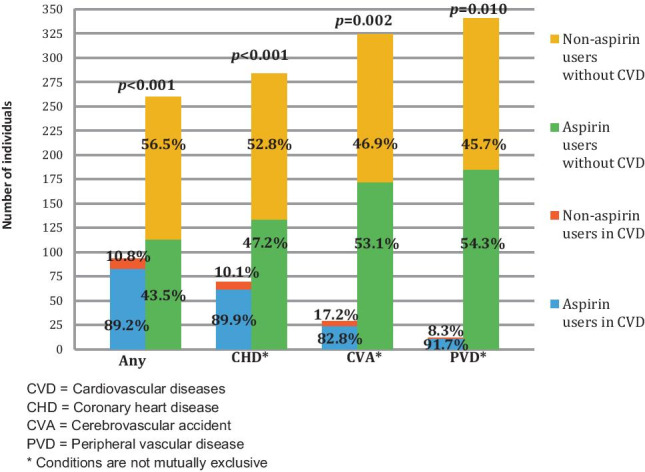
Aspirin use in participants with cardiovascular disease (excluding oral anti-coagulants). CVD cardiovascular diseases, CHD coronary heart disease, CVA cerebrovascular accident, PVD peripheral vascular disease. *Conditions are not mutually exclusive

Forty-seven non-aspirin users who were on other antiplatelet agents/anticoagulants were excluded from the analysis for non-aspirin use, as their use would obviate the need for aspirin. The distribution of aspirin use among patients with CVD is shown in Fig. 2. The use of aspirin among PVD patients was significantly higher than any other group, with 91.7% patients on aspirin.

**Fig. 3:**
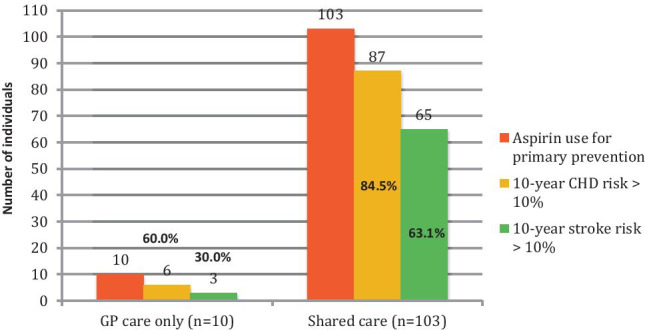
10-year cardiovascular risk in participants taking aspirin for primary prevention. CHD coronary heart disease

Of all participants who were prescribed aspirin, 57.7% were using it for primary prevention, and 42.3% were on it for secondary prevention. About 91.2% of participants in primary prevention for CVD category were under shared care. The 10-year cardiovascular risk calculated using the UKPDS risk engine for patients under primary prevention is shown in Fig. 3. In addition, the study demonstrated that participants in the secondary prevention of CVD group were significantly older (*p* = 0.001). There was no significant difference in terms of diabetes control and cardiovascular risk factors between the two prevention groups.

## Discussion

In this study, 49.0% of the population was prescribed aspirin, among whom 86.2% were attending shared care. However, despite such a high prevalence of CVD and the international consensus for aspirin use as secondary prevention for CVD, [13, 14, 16] 10.8% of individuals with CVD, who were attending shared care, were not on aspirin or any other antiplatelet/anticoagulant. This may be due to potential contraindications to aspirin use, such as previous gastrointestinal bleeding, history of peptic ulcer disease, or possible medication interactions.

Primary prevention with aspirin has always been controversial. Multiple randomized controlled trials (RCTs) were conducted on aspirin use in primary prevention of CVD over the past 30 years, notably the British Male Doctors Study (1988), [17] Physicians’ Health Study (1989), [18] Early Treatment Diabetic Retinopathy Study (1992), [19] Hypertension Optimal Treatment randomised trial (1998), [20] Thrombosis Prevention Trial (1998), [21] Primary Prevention Project (2001), [22] and Women's Health Study (2005), [23] among others. A meta-analysis of four of the mentioned RCTs reported a significant reduction of all cardiovascular events by 15.0%, and myocardial infarctions by 30.0%. However, this was balanced with an increased risk of bleeding by 69.0%. [24] Two other meta-analyses done in 2011 reported similar findings—a decrease in total cardiovascular events, and non-fatal MI, but no statistical reduction in stroke, or all-cause mortality. [25, 26] Berger et al. [26] also concluded that over a mean follow-up of 6.9 years, the number needed to harm was 1 major bleed in 261, which counterbalanced the number needed to treat (253 patients), in order to prevent 1 major cardiovascular event.

A systematic review including all of the 11 RCTs aforementioned noted a 14.0% reduction in nonfatal stroke with the use of ≤ 100 mg aspirin, on top of the well-established nonfatal MI decline. However, despite the addition of 18,369 participants in two other RCTs, the decrease in all-cause, and cardiovascular mortality remains insignificant. [27] The most recent ASCEND trial comparing 15,480 randomised patients with diabetes on either 100 mg aspirin daily or placebo, suggested similar findings. The benefits of CVD risk reduction (8.5% vs 9.6%; *p* = 0.01) was counterbalanced with major bleeding risks (3.2% vs 4.1%, *p* = 0.003). [11] These results have been further borne out in the ASPREE study and the ARRIVE trial. Both of these studies excluded diabetic patients but still demonstrated risk: benefit neutrality for aspirin therapy without confirmed vascular disease^.^ [9, 10].

As of 2020, aspirin use for primary prevention is no longer recommended by most guidelines, [14,16] except for the ADA which advise *consideration* of use for individuals with > 10% of 10-year cardiovascular risk. [13] Our study documented that 40.0% and 70.0% of primary prevention persons under GP care only had less than 10% risk of 10-year CHD risk and 10-year stroke risk, respectively. Similarly, 15.5% and 36.9% of the shared care participants had less than 10% risk of 10-year CHD, and 10-year stroke risks, respectively (Fig. 3). According to the ADA guideline, [12] these individuals should not be prescribed aspirin. It was suggested that doctors tend to miscalculate individuals’ CVD risk due to the lack of universal risk assessment tools or calculators. [28] Discrepancies in guidelines may result in confusion for physicians, and physicians may also be unlikely to discontinue a medication prescribed by other healthcare professionals.

Upon review of the available literature to date, it is imperative to note that an increased risk for CVD in individuals with type 2 diabetes should not mandate aspirin use. Healthcare professionals are required to perform a thorough risk assessment tailored to each individual before prescribing aspirin therapy. Type 2 diabetes mellitus is associated with a greater number of comorbidities and complications, which inevitably, increase the cost of management. An Irish study done in 2010 indicated that persons with type 2 diabetes ≥ 65 years who were covered by the General Medical Services scheme had a higher average annual pharmaceutical cost for management of comorbidities (€1238.67) in contrast to those without diabetes (€799.28). [29] Therefore, patient education and strict adherence to therapeutic guidelines are recommended to prevent complications, and thereby also reduce costs to the healthcare system. Evidence-based indications should be implemented to improve appropriate drug usage, including aspirin, in individuals with diabetes. In addition, these individuals should have their medications reviewed thoroughly every year.

Some limitations of this research include the retrospective design of the study, incomplete data from clinical case notes, and measurement of the most recent, single clinical and laboratory data, which could result in over- or under-diagnoses. Aetiology of stroke was important as haemorrhagic stroke would preclude the need for aspirin, but it was not differentiated in this study. In addition, most studies adopted the Framingham risk score for 10-year CVD risk or the QRISK2 score, while this study used the UKPDS risk engine, as the latter is tailored for diabetes patients. A larger sample size and a longer study period would be recommended to provide better statistical results. A larger geographical catchment area also should be considered for future studies.

In conclusion, type 2 diabetes and CVD account for the most non-communicable diseases’ deaths globally. Therefore, achievement of therapeutic targets for individuals with type 2 diabetes should be monitored closely and improved in order to reduce cardiovascular risk. A significant proportion of persons with diabetes should be, but were not, prescribed aspirin for secondary prevention. This is in contrast to the substantial population of those who should not be, but were on aspirin for primary prevention in this study. Both primary care and secondary care health professionals should re-evaluate their aspirin prescription among individuals with type 2 diabetes to ensure the utmost benefits are achieved.
